# Fertilizer management practices in potato cultivation: a baseline study for the introduction of GE potato in Bangladesh

**DOI:** 10.3389/fbioe.2024.1409996

**Published:** 2024-07-12

**Authors:** Abu Shamim Mohammad Nahiyan, Saiful Islam, Aparna Islam, Mohammad Ataur Rahman, Mohammad Mahmood Hasan, Tasnin Khan Eusufzai, Mohsina Afreen, Fareyzul Haque Ansarey, Tahmina Khan, A. F. M. Jamal Uddin

**Affiliations:** ^1^ Advanced Seed Research and Biotech Centre, ACI Limited, Dhaka, Bangladesh; ^2^ National Key Laboratory for Germplasm Innovation & Utilization of Horticultural Crops, Huazhong Agricultural University, Wuhan, China; ^3^ Biotechnology Program, Department of Mathematics and Natural Sciences, BRAC University, Dhaka, Bangladesh; ^4^ Department of Microbiology and Cell Science, University of Florida, Gainesville, FL, United States; ^5^ Department of Horticulture, Faculty of Agriculture, Sher-e-Bangla Agricultural University, Dhaka, Bangladesh

**Keywords:** agronomic practices, baseline data, biosafety assessment, fertilizer management, potato

## Abstract

Genetically engineered (GE) crops have the potential to contribute to agricultural sustainability, food security, and nutritional enrichment. However, these crops cannot be released for commercial cultivation without undergoing environmental risk assessments (ERA), thus biosafety evaluation. ERA assessments are performed comparatively with their natural non-GE counterparts. As Bangladesh is progressing with GE potato research, the present study aims to collect baseline information on non-GE potato cultivation with an emphasis on current agronomic practices focusing on fertilizer management and farmers’ knowledge base. The survey had three parts, including information on the farmers, information on potato cultivation practices, especially fertilizer use, and lastly, the farmer’s view on GE potato. From 2020 to 2021, data were collected through interviews with experienced growers in four potato-growing regions, the Central and Mid-East, North-West, Mid-West, and South-East regions (n = 1757) of the country. The study revealed that farmers of all regions used more than the recommended amounts of fertilizer; for instance, 67.1% more nitrogen fertilizer was applied as an extra dose during potato cultivation in Munshiganj (Central and Mid-East) than in the Dinajpur region (North-West). This overuse of nitrogen fertilizer can enhance plant vigor but makes the plants more susceptible to insect attraction and allows pests easier access to the plants. As a result, the excess dose of nitrogen fertilizer in Munshiganj may act as a catalyst to increase the probability of late blight. The findings also showed that 73.6% of farmers observed unexpected flowering in certain potato cultivars, which corresponded to the higher application of phosphate and potassium fertilizers aimed at late blight control. Furthermore, this study reported infestations of Solanaceous weeds, specifically *Solanum torvum* and *Physalis heterophylla*, in potato fields. Finally, our findings demonstrated that more than 68.7% of the potato growers intend to adopt disease-resistant GE potato as that may reduce the need for excess fertilizer use and thus reduce cultivation costs.

## 1 Introduction

Potato (*Solanum tuberosum*), belonging to the family Solanaceae, is a staple food and the world’s fourth most important crop after rice, wheat, and maize (Islam et al., 2017). Bangladesh ranks as the seventh largest potato-growing country globally and third-largest in Asia (Crops. FAOSTAT, 2018). Recent data indicate that Bangladesh is self-sufficient in potato production, yielding 9.87 million metric tons by cultivating 468.7 thousand hectares of land (BBS, 2023). Despite this impressive production, potato exports are significantly hindered due to their compromised quality, which results from various diseases (Mittler, 2006).

Potato is highly susceptible to various diseases caused by fungi, viruses, and bacteria, resulting in significant crop damage and yield loss (Islam et al., 2022). Farmers commonly use fungicides and insecticides to combat these infestations (Kromann et al., 2009). However, the extensive use of these chemicals raises serious food safety and environmental concerns, highlighting the need for alternative disease management strategies (Islam et al., 2018).

Plant breeders have worked relentlessly for centuries to develop improved crop varieties with enhanced disease resistance. Although most disease-resistant crops have been developed through conventional and molecular breeding approaches, these methods have limitations, for instance, limited genetic resources within the gene pool, cross-incompatibility, and prolonged development phases. Genetic engineering can overcome these barriers by allowing the integration of beneficial genes from any source into targeted crops, providing tolerance and resistance against both abiotic and biotic stresses in a shorter breeding time. Moreover, considering the adverse impacts of chemical treatments, the introduction of disease-resistant potato varieties through genetic engineering offers a feasible and environment-friendly alternative for disease control (Lozoya-Saldana, 2011). Experience from nearly 3 decades of commercial cultivation of various GE crops has demonstrated its benefits to the growers. Therefore, the adoption of GE crops is rising globally (Bhajan et al., 2022), particularly in developing countries like Bangladesh (James, 2017).

In 2013, Bangladesh became a biotech country by adopting Bt eggplant, a genetically modified variety developed by introducing the Cry1Ac gene from *B. thuringiensis*, which provides resistance against eggplant fruit and shoot borer (EFSB) (Shelton et al., 2018). In 2023, commercial cultivation of Bt cotton began, which was developed by inserting the same gene of the Cry family (*Cry1AC*) as Bt brinjal from *Bacillus thuringiensis*, conferring resistance to cotton bollworm (ISAAA, 2023; Sujan et al., 2024). Bangladesh has been engaged in research on GE potato since 2006, and to date, several confined field trials were conducted on late blight (LB) resistant potato developed by introgression of three LB resistance genes, *Rpi-mcq1, Rpi-blb2, and Rpivnt1.1* (Potatopro, 2021; USDA, 2021).

However, the commercial cultivation of GE crops requires prior biosafety assessments to ensure environmental safety. According to the Guidelines for the Environmental Risk Assessment (ERA) of Genetically Engineered (GE) Plants 2016, one key issue to consider is the comparison of cultivation practices between GE and non-GE natural counterparts. Agronomic performance and crop physiology also need to be evaluated during these assessments. Therefore, it is necessary to understand the common cultivation practices employed by Bangladeshi farmers for potato cultivation and to identify potential changes that may occur with the introduction of the GE potato.

Considering the above-mentioned issues, a foundational survey was conducted throughout the potato-growing season to collect information from both potato growers and seed potato producers. The study aimed to understand their approaches to fertilizer management and other agricultural practices during potato cultivation. To achieve this goal, several specific objectives were outlined, including i) Collecting baseline information on potato cultivation, ii) Gathering data on the use of macro and micronutrients in the form of manures and fertilizers in potato cultivation, and iii) Understanding the attitude of Bangladeshi potato growers toward GE potato.

## 2 Materials and methods

### 2.1 Demographic site selection

**FIGURE 1:**
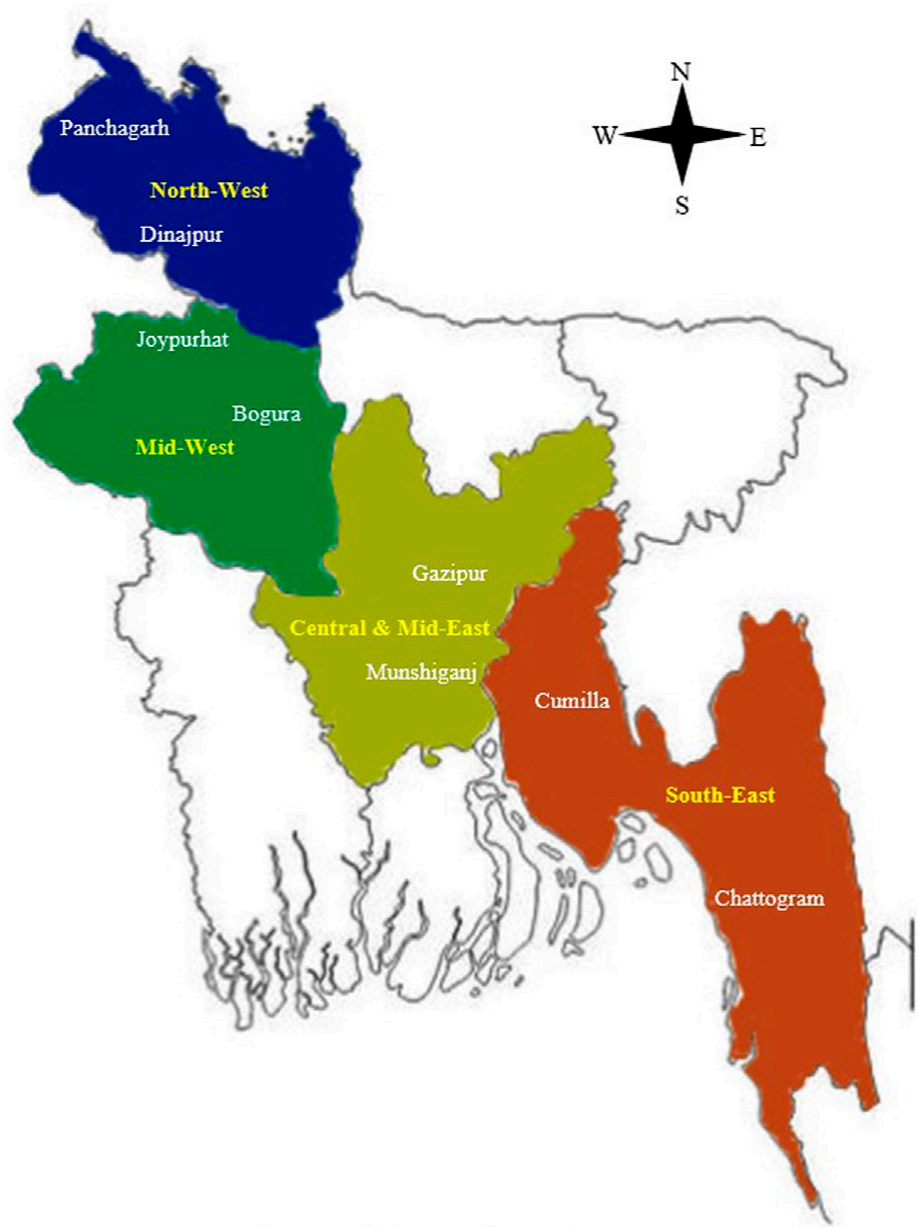
Geographic distribution of survey sites across Bangladesh, encompassing four study regions.

A total of 1,757 potato growers were randomly interviewed across various upazilas, taking into account the diversity in climate and soil types across regions, with consideration given to their varying levels of academic knowledge and financial circumstances. A total of 16 study locations have been selected from the four major potato-growing regions in Bangladesh, namely, i) Central and Mid-East (Munshiganj and Gazipur districts, n = 404), ii) North-West (Panchagarh and Dinajpur districts, n = 527), iii) Mid-West (Joypurhat and Bogura districts, n = 439), and iv) South-East (Cumilla and Chattogram districts, n = 387) (Figure 1). Each region was represented by two districts, and within each district, two upazilas were chosen for data collection and analysis.

### 2.2 Survey

This survey was carried out by the Advanced Seed Research and Biotech Centre (ASRBC), ACI Ltd., Dhaka, Bangladesh. In this survey, information and data were collected from farmers owning at least 10 decimals (1 decimal = 435.56 sq. feet) of land for potato cultivation. The potato growers from each region were selected through a standard field survey technique (BBS, 2014). The risk impacts survey consisted of five parts: 1) fertilizer management, 2) agronomic practices, 3) disease and insect infestation, 4) weed infestation, and 5) knowledge and willingness about GE technology among farmers.

### 2.3 Data collection methods

**FIGURE 2:**
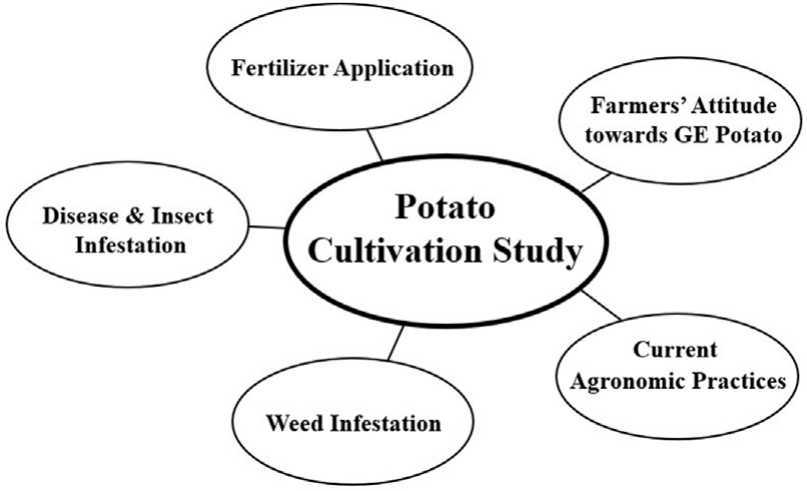
Research model of the study.

The data collected through a pre-tested questionnaire were tabulated, processed, and analyzed, keeping in view the goal and objectives of the study. Information and data were collected by a team of 16 designated enumerators, with two enumerators assigned to each district, as part of this comprehensive survey conducted among farmers. The questionnaire included several key data indicators, encompassing the farmer’s demographic information such as name, age, sex, and education, as well as details regarding farm size, agricultural practices, and the management of manures and fertilizers. Additionally, the questionnaire also addressed the recommended doses of fertilizer, potential risks, and economic damages stemming from insect or pest infestations in potato cultivation. It also inquired about the effectiveness of the farmers’ weed, insect, or pest control measures and assessed their attitudes toward acceptance of advanced technology for potato production. A theoretical working model (Figure 2) was utilized that integrates the innovation adoption model by Rogers et al. (2003) with the construct of attitude from the attitudinal models by Fishbein and Ajzen (1975), as outlined in the research model proposed by Kwade et al. (2019). This approach enabled us to identify factors related to agronomic practices in potato cultivation, explore the risks associated with potato farming, and examine the attitudes of the people of Bangladesh toward genetic engineering (GE) technology.

### 2.4 Statistical analysis

To evaluate the association between attitudinal variables, bivariate Pearson correlation analysis was used with the help of GraphPad Prism 9.00, United States. A paired sample *t*-test was used to examine the differences between the opinions. Multinomial logistic regression was used to identify significant variables of growers’ perceptions of major obstacles in potato production and willingness to adapt GE technology for potatoes. As a result of allocating the response categories, the dependent variable was transformed into a variable with two categories (yes/no). This strategy was used because our primary objective was to estimate the likelihood that people will accept the GE potato (willing or not) while also allowing for the assessment of differences between the indifferent farmers and the intended adapters or non-adapters. Because of the strong correlation between farm size (ha) and potato cultivation area (ha), the latter was not included in the model. Gender was excluded from the socio-demographic variables due to its extremely skewed distribution toward male farmers. Knowledge of GE technology, ethical and socio-economic concerns, and environmental benefits were entered as continuous variables in the model, whereas farm size, farmer education level, number of fertilizer applications, age, and experience in potato cultivation were included as dummy variables. The demographic information about participants (n = 1757) is shown in Table 1, and the questionnaire containing the statements chosen for data collection and analysis is shown in Table 2.

**TABLE 1:** Gender, age group, and academic qualification of the study population (n = 1757).

Demographic profile	Category	Number	Percentage (%)
Gender	Male	1729	98.40
	Female	28	1.60
Age group	18–20 years	26	1.50
	21–30 years	504	28.70
	31–40 years	761	43.30
	41–50 years	365	20.80
	Over 50 years	101	5.70
Academic qualification	Elementary school	763	44.80
	Secondary school	543	30.90
	Higher secondary school	390	19.20
	University	61	5.10

The demographic characteristics of the study participants, detailing their gender distribution, age groups, and academic qualifications, are presented. The total sample size was 1757 individuals.

**TABLE 2:** Selected statements on common agronomic practices during potato cultivation in Bangladesh according to the questionnaire.

Statement
1. Both organic (cow dung and ash) and inorganic (chemical fertilizers) fertilizers are frequently used in potato cultivation across the country 2. Basal dosage as the granular form is used to apply TSP (P), magnesium (Mg), gypsum (S), and boron (B), except urea (N) and MOP (K) fertilizers, during the last plowing of the soil preparation 3. Urea and MOP are applied as split doses in the first and second earthing up of potato cultivation 4. Micronutrients: boron (B), copper (Cu), zinc (Zn), and manganese (Mn) are supplied in liquid form 5. High dependence on fertilizers in potato cultivation is not only for plant growth enhancement but also for disease management 6. Several weeds (*S. torvum*, *P. heterophylla*, *A. philoxeroides*, *C. album,* and *A. viridis*) have been identified in potato fields and are primarily managed manually 7. Groundwater dependency for irrigation is very high 8. The advanced agronomic practices that are employed in quality potato production (processing and export quality) globally are largely unknown to farmers 9. After harvesting, plant debris is left in the field 10. Late blight, virus infestation, and scab disease are the most common diseases that significantly affect potato quality and yield in the country 11. Excessive pesticides and insecticides are frequently used to control diseases 12. Farmers have no knowledge about environmental pollution when applying fertilizers and pesticides in the potato field 13. Positive attitudes have been shown among the farmers in the acceptance of disease-resistant and high-yielding GE potato varieties

These statements from the questionnaires reflected common agronomic practices employed during potato cultivation in Bangladesh. These statements are designed to capture key aspects of agricultural methods and techniques used by local farmers.

## 3 Results

The findings of the survey have been categorized and presented in several ways to demonstrate the current state of potato production as well as potential strategies of fertilizer management and agronomic practices for the introduction of GE potato in Bangladesh.

### 3.1 Demographic information

According to the demographic profile of the respondents (n = 1757), the sample consisted of a group of participants with characteristics that reflected the diversity of the research population (Table 1). The gender distribution was 98.4% male and 1.6% female, reflecting the typically male-dominated agricultural profession. Most of the total study population had more than 10 years of experience in cultivating potato (data not shown). In addition, the largest age group of participants, at 43.3%, was people between the ages of 31 and 40 years. In terms of formal education, only 5.1% of the participants held a college-level degree, while 44.8% had only elementary education.

### 3.2 Responses to the statements

In the designated four regions, respondents were interviewed face-to-face regarding the current state of potato production technology, fertilizer management practices, disease and weed infestation, and their attitudes toward adopting GE crops. During the interviews, 13 statements were selected from the questionnaire to reflect the specific aims of the proposed study (Table 2). Most farmers in the survey regions followed conventional fertilizer application strategies (statements 1–5). During land preparation, they applied both organic and inorganic fertilizers as basal doses. Additionally, liquid fertilizers were often applied during the growing stages to address macro- and micronutrient deficiencies. Weed diversity is a vital cause of yield reduction and was managed manually to maintain the nutritional balance in potato plants (statement 6). None of the farmers expressed concern about the biosafety issues that could arise from plant debris residues during haulm pulling or potato harvesting (statement 9). Furthermore, most participants expressed positive attitudes toward adopting disease-resistant genetically engineered (GE) crops, which aim to reduce yield losses due to pests. This adoption would help address challenges in disease management and production losses, thereby supporting farmers in generating higher profits.

### 3.3 Assessment of the existing fertilizer management scenario

Generally, farmers in Bangladesh depend on the traditional system of potato cultivation. They follow the recommended doses of fertilizer outlined by Azad et al. (2019) to achieve a significant increase in production (Table 3). Nonetheless, during the survey, it was observed that the major fertilizers, such as urea (N), TSP (P), and MOP (K), were applied excessively in potato cultivation. In the Central and Mid-East region, nitrogen fertilizer was applied at 134.0% of the recommended dose, amounting to 250 kg/ha. Whereas in the North-West, phosphorus was applied at 163.3% of the recommended dose, and potassium was applied at 117.2% of the recommended dose. The recommended amounts are 150 kg/ha and 250 kg/ha, respectively.

**TABLE 3:** Fertilizer management practices of the four regions compared to the recommended dose for potato cultivation in Bangladesh.

Fertilizer	Recommended dose (kg/ha)^a^	Central and Mid-East (kg/ha)	North-West (kg/ha)	Mid-West (kg/ha)	South-East (kg/ha)
Urea	250	585	350	360	385
TSP	150	298	395	325	243
MOP	250	385	543	487	363
Gypsum	120	115	121	119	118
Zinc sulfate	10	10	10	11	10
Magnesium sulfate	100	95	98	100	112
Boric acid	10	9	8	10	10
Cow dung	9,071	4,580	5,690	4,500	5,600

^a^ Recommended doses reported by Azad et al., 2019 in the *Krishi Projukti Hatboi* (*Handbook on Agro-Technology*). Published by the Bangladesh Agricultural Research Institute (BARI).

Comparing these fertilizer management practices across four different regions with the recommended fertilizer doses highlighted variations in nutrient application and adherence to agricultural guidelines during potato cultivation in Bangladesh.

**FIGURE 3:**
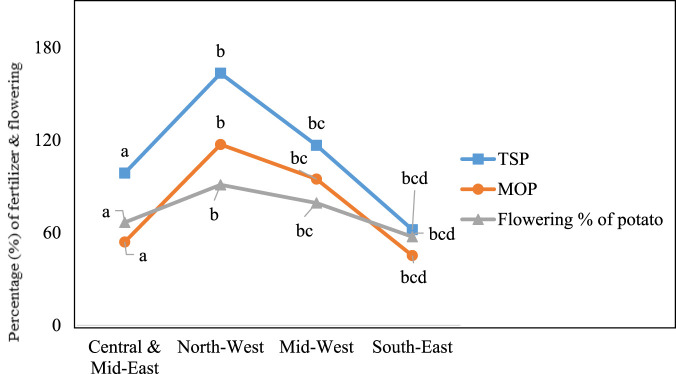
Induced flowering (%) in potato resulting from overuse of TSP and MOP across the four study regions; ^abc^ indicates a significant difference at *p* = 0.05 among the regions.

**FIGURE 4:**
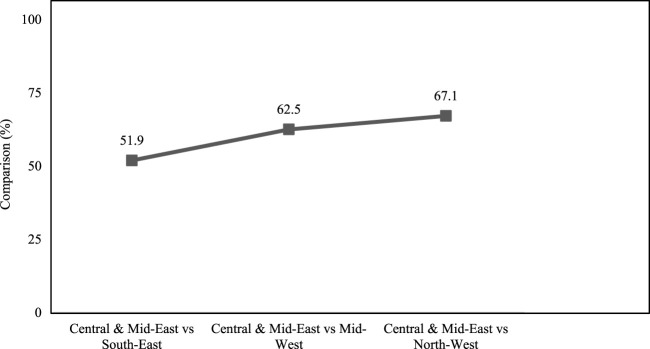
Comparison (%) between Central and Mid-East with the other three regions in the case of excessive urea application during potato cultivation.

In addition, significant differences were found in the excessive application of TSP and MOP during potato cultivation among the four studied regions. During cultivation, early physiological maturity of potato plants followed by induced flowering was observed in these fields. Among our interviewees, 73.6% of farmers reported observing unexpected flowering in some potato varieties when they applied more TSP and MOP fertilizer than the recommended dose to control late blight disease (Figure 3). On the other hand, the potato growers of Central and Mid-east assumed that the excessive use of urea may increase the potato yields. The line graph (Figure 4) illustrates the relationship between the percentage of excess urea usage in the Central and Mid-East region compared to the other three studied regions, namely, the South-East, the Mid-West, and the North-West region. The data revealed that the difference in urea application between the Central and Mid-East region and the South-East, at 51.9%, is the lowest. In contrast, the discrepancy in urea usage between the Central and Mid-East and the North-West was the highest at 67.1% (Figure 4).

### 3.4 Obstacles to potato cultivation

A significant portion of the interviewed farmers identified various diseases, such as late blight, virus infection, scab, etc., as the most severe challenge in potato cultivation, leading to substantial crop damage and yield loss. In the North-West, approximately 35.4% of farmers reported significant yield loss and quality deterioration due to these diseases, while 32.7% of farmers in the Mid-West experienced similar issues. Our survey found that late blight was the most devastating disease, reported by approximately 10.7%–19.1% of respondents (Table 4). Approximately 23.0% of respondents reported insect and pest attacks in the North-West region, and 15.25% of respondents in the Mid-West identified weed infestation as a major problem in their potato fields. Moreover, due to adverse calamity, excessive rainfall was also observed in the dry season by 2.2%–7.4% of the farmers. This rainfall caused severe water management problems followed by crop damage due to disease outbreaks, especially late blight. Additionally, inadequate storage facilities were reported by 3.1%–11.9% of respondents; inadequate storage causes massive tuber loss by different fungal and bacterial infections. Moreover, due to rising costs and the scarcity of fertilizers and insecticides in the market, farmers struggled to grow potatoes profitably.

**TABLE 4:** Growers’ perceptions (%) of major obstacles observed in potato production across the four study regions.

Obstacle	Response (%) to major obstacles				
	Central and Mid-East	North-West	Mid-West	South-East	LSD
1. Disease infection	29.00 ± 0.447^a^	35.40 ± 0.435^b^	32.69 ± 1.249^bc^	23.57 ± 1.086b^cd^	2.536
a. Late blight	12.50	19.10	15.80	10.75	
b. Virus	9.40	11.60	10.30	7.60	
c. Scab	7.10	4.70	6.59	5.22	
2. Insect attack	14.62 ± 0.388^a^	23.00 ± 0.471^b^	19.00 ± 0.453^bc^	11.70 ± 0.494^bcd^	1.301
3. Weed infestation	9.35 ± 0.212^a^	11.25 ± 0.700^b^	15.25 ± 0.366^bc^	8.50 ± 0.500^a^	1.375
4. Lack of irrigation facilities	8.40 ± 0.600^a^	7.00 ± 0.391^b^	4.20 ± 0.493^bc^	5.30 ± 0.251^bc^	1.298
5. Pest attack in storage	13.80 ± 0.872^a^	9.10 ± 0.524^b^	10.70 ± 0.626^b^	10.75 ± 0.656^b^	1.956
6. Excessive rainfall	2.16 ± 0.314^a^	4.75 ± 0.395^b^	3.56 ± 0.382^bc^	7.36 ± 0.423^bcd^	1.093
7. Lack of storage facilities	8.80 ± 0.694^a^	3.10 ± 0.202^b^	4.70 ± 0.394^b^	11.90 ± 0.908^bc^	1.758
8. Lack of accuracy in pricing	9.89 ± 0.613^a^	2.00 ± 0.168^b^	5.50 ± 0.526^bc^	13.96 ± 0.817^bcd^	1.666
9. Problems to collect agrochemicals	0.50 ± 0.092^a^	3.80 ± 0.354^b^	1.80 ± 0.129^bc^	2.87 ± 0.316^bcd^	0.719
10. Others	3.48 ± 0.127^a^	0.60 ± 0.115^b^	2.60 ± 0.214^bc^	4.09 ± 0.259^bcd^	0.542

^abc^ indicates a significant difference at *p* = 0.05 among the regions.

The data show the percentage distribution of growers’ perceptions of major obstacles in potato production across four study regions, highlighting regional differences and localized issues. Significant differences were observed among the regions at *p* = 0.05.

### 3.5 Weed diversity

**FIGURE 5:**
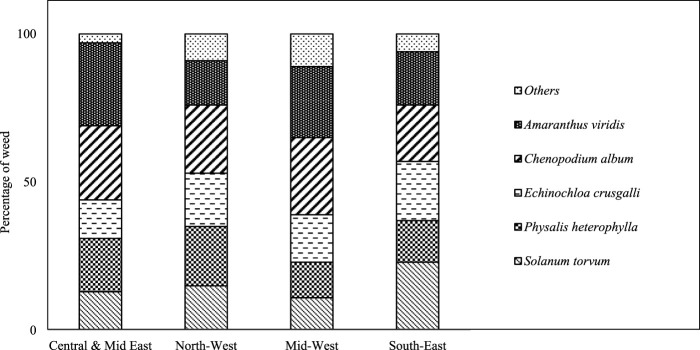
Weed diversity during potato cultivation among the four study regions.

**FIGURE 6:**
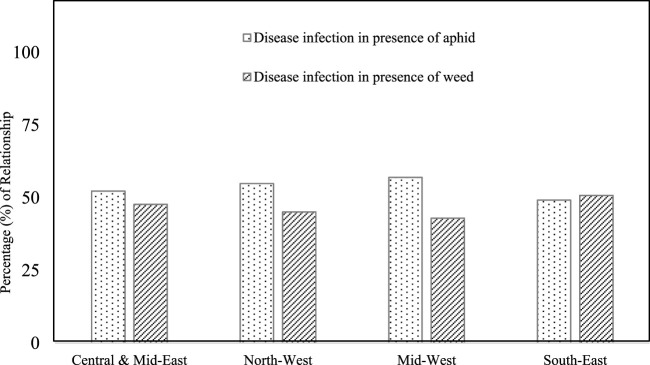
Relationship (%) between aphid and weed infestation in potato fields across the four study regions.

The farmers mentioned weed infestation in their potato fields. *S. torvum*, *P. heterophylla*, *Alternanthera philoxeroides*, *C. album,* and *Amaranthus viridis* weeds are generally found in potato fields in Bangladesh (Figure 5). Farmers observed weeds in their potato fields at approximately 30 DAP (days after planting). *Solanum torvum*, *Physalis heterophylla,* and *A. philoxeroides* are from the Solanaceae family. *Chenopodium album* and *A. viridis* are from the Amaranthaceae family. Among the five mentioned weeds, *A. viridis* (28%) and *S. torvum* (23%) were found to have high densities in the Central and Mid-East and South-East regions compared to the other regions. These weeds significantly reduce productivity by competing with potato crops for nutrients. Respondents from the North-West and Mid-West regions reported the existence of vectors, namely, aphids that cause rapid virus transmission, while weed infestation was also observed (Figure 6). These reports indicate there was a strong and positive association between pests and weed infestation.

### 3.6 Willingness to adopt GE crops

**FIGURE 7:**
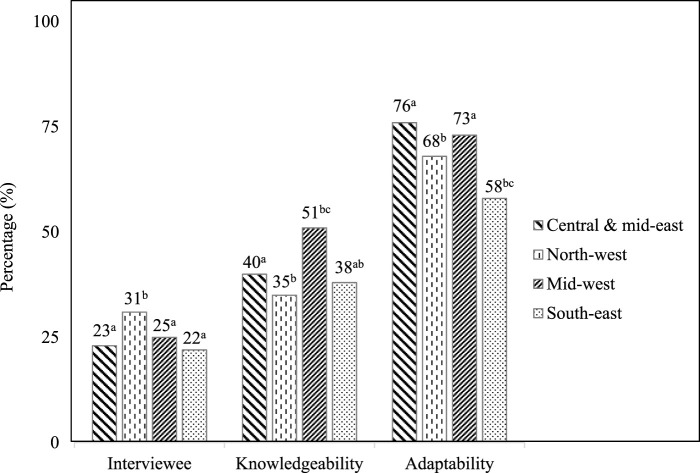
Willingness to adopt GE technology among farmers in the four study regions (^abc^ indicates a significant difference at *p* = 0.05 among the regions).

The knowledge and keenness to accept the GE technology were evaluated among the participants interviewed, as illustrated in Figure 7. In this study, we observed that more than 50% of interviewees were willing to integrate GE potatoes into their potato cultivation system as they think GE crops will have high yield potential and their disease resistance may also reduce cultivation costs. The Central and Mid-East region showed the highest enthusiasm for adopting GE technology, with 76% of participants expressing willingness, while the South-East region had the lowest at 58%.

## 4 Discussion

The statistical populations for this survey in the four regions were mostly regular farmers with more than 10 years of experience. Respondents (n = 1757) were grouped based on their gender, age, and educational qualifications, as reported by De Steur et al. (2019) (Table 1). To ensure the effectiveness of the study, a purposeful random sample technique was utilized to choose respondents from various regions. This approach aimed to determine fertilizer management procedures, cultural practices, and farmer attitudes toward genetically engineered (GE) potato, which was reflected through the 13 statements in Table 2. This methodology was supported by Areal et al. (2011), who summarized farmers’ attitudes toward genetically modified herbicide-tolerant crops with 24 statements.

Though Bangladesh produces a huge amount of potato, the quality of potato has undergone several challenges due to biotic and abiotic stresses. In terms of the use of fertilizers, we have recorded that all of the interviewed farmers applied more fertilizer than the recommended dose with an expectation of higher yield and better disease management (Table 3). Similar phenomena were observed in a study where the farmers of Munshiganj applied 3–4 times higher doses of urea, TSP, and MOP fertilizers than was recommended for a potato field (Choudhury et al., 2006). We observed that this attitude was more frequent among farmers who did not have a high school or college diploma. Due to a lack of proper education and understanding of fertilizers, including their mode of action, most of the farmers in Bangladesh believe that a higher application would benefit them with a higher yield and, thus, more profit.

Consequently, in this survey, 73.6% of farmers reported that an extra amount of TSP and MOP fertilizer could reduce the devastating damage to potatoes by controlling late blight diseases in the North-West and Mid-West regions. The high cost of pesticides or herbicides might be one reason behind the using extra fertilizers to tackle pests and pathogens. Apart from this, potato plants that received an excessive amount of TSP and MOP for a prolonged period experienced an unexpected flowering and physiological maturity compared to plants that received the recommended rate of fertilizer (Figure 3). The result of this survey is consistent with that of Setu et al. (2018), who observed the same effect of TSP and MOP. They reported these fertilizers did not affect potato phenotype, growth parameters, or tuber yields; however, they had a significant impact on days to unexpected flowering, physiological maturity, plant height, leaf area, above and underground dry biomasses, and marketable yield.

In addition to TSP and MOP, we observed that 67.1% of excess urea as a nitrogen source was applied in Munshiganj (Central and Mid-East) compared to Dinajpur (North-West) during potato cultivation. Moreover, a relationship between the percentages of excessive use of urea in the Central and Mid-East region compared with three other regions, namely, the South-East, the Mid-West, and the North-West regions, was studied. Given that the recommended dose of urea application was 250 kg/ha, each region was noted to overuse this fertilizer, especially the Central and Mid-East region. Excessive amounts of urea produce more succulent vegetative parts, leading to increased plant vigor with darker green coloration, which in turn attracts more insects and pests (Singh and Sarkar, 2021). This may act as a catalyst to increase the probability of late blight and boost insect infestation and virus infection.

Apart from the disease infestations, several other obstacles were recorded during potato cultivation, including the absence of suitable germplasm, difficulties in water management, lack of storage facilities, unexpected weather, etc. (Table 4). Similar obstacles were also reported during potato cultivation in Bangladesh by Hoque et al. (2023). In the case of water management, farmers faced obstacles to managing groundwater for irrigation and unexpected rainfall, which directly hampered the yield. Similarly, Sun et al. (2015) found that the frequency of watering at different phases of potato growth and development impacts the production and quality of the crop.

Weeds also pose significant challenges in potato production in Bangladesh through nutrient uptake and playing host to pathogens (Showler and Granberg, 2003; Faruq et al., 2021). Potato yield reduction due to weed competition was extensively documented by Rana et al. (2004) and Attri et al. (2022). The present study observed that *S*. *torvum*, *P*. *heterophylla*, *A*. *philoxeroides*, *C*. *album,* and *A*. *viridis* are some of the weed species in potato fields. Similarly, Khan et al. (2008) demonstrated that *C*. *album* and *A*. *viridis* are the main weed species in the potato fields of Bangladesh. Cuda et al. (2002) and Khokon et al. (2017) showed that *S*. *torvum* and *P*. *heterophylla* are major weeds of the Solanaceae family. Moreover, many insects, including aphids, thrips, whiteflies, and leafhoppers, transmit plant diseases from weeds (Capinera, 2005). Future studies may be needed to assess the potential outcrossing among these weeds and potato.

The current survey aimed to study the agronomic practices of potato cultivation in Bangladesh along with the effect of fertilizer on crop performance, the weed population, and overall challenges during cultivation. The final goal is to understand farmers’ willingness to adopt GE potato. The study reveals a willingness among farmers to adopt genetically engineered (GE) potatoes. However, the acceptance level varies among the study regions. Several studies have shown that the variation in acceptance due to the presence and absence of effective science communication about the benefits (Farid et al., 2020; Abdul Aziz et al., 2022), cultural attitudes, support systems, and local agricultural services (Koralesky et al., 2023). In the future, the reason for the variation in the study regions may be assessed.

## 5 Conclusion

The study was conducted in four major potato cultivation areas of Bangladesh. Farmers with more than a decade of experience in potato cultivation were interviewed. The growers’ responses showed that fertilizers are usually applied at higher-than-recommended doses to ensure optimum yield while controlling disease infestation by making the plants vigorous. This overdose was found to correspond to early flowering in the field. Common weed populations were also recorded in the study. Several cultivation challenges, including diseases and pest infestations, were mentioned in response to the questions asked during the survey. A large percentage of farmers opined in favor of the cultivation of GE potato due to their understanding that it will give higher yield and disease resistance. However, the questions also revealed their lack of in-depth knowledge of GE crops. The present study deals with farmers’ agronomic practices and observations about potato cultivation. This can partially fulfill the required baseline data for ERA prior to the introduction of GE potato in Bangladesh.

## Data Availability

The raw data supporting the conclusions of this article will be made available by the authors, without undue reservation.
